# The efficacy of bactrim in reducing surgical site infections after spine surgery

**DOI:** 10.1016/j.xnsj.2021.100095

**Published:** 2021-12-06

**Authors:** Jeffrey Hyun-Kyu Choi, Huy Alex Duong, Sean Williams, Joshua Lee, Michael Oh, Charles Rosen, Yu-Po Lee, Nitin Bhatia

**Affiliations:** a Department of Orthopaedic Surgery, University of California, Irvine, CA, United States; b Department of Neurosurgery, University of California, Irvine, CA, United States

**Keywords:** Surgical site infection, Gram negative, Bactrim, Lumbar fusion, Spine surgery

## Abstract

**Background:**

Previous studies show an increasing incidence of gram-negative organisms in surgical site infections after spine surgery. This study is looking for the association of the post-operative prophylactic use of Bactrim and the gram-negative surgical site infection after lumbar spine surgery

**Methods:**

Patients who underwent lumbar spine surgery between August 2010 and December 2019 at the institution were retrospectively reviewed.

**Results:**

There were 11 infections out of 511 cases where no oral antibiotics were given (2.2%). There were 2 infections out of 84 cases where Bactrim was given (2.4%). This was not statistically significant (P=0.89). The organisms cultured from the no oral antibiotic group were 8 cases of methicillin sensitive Staphylococcus aureus (MSSA), 1 case of E. coli, 1 case of Pseudomonas aeruginosa, 1 case of MRSA. The organisms cultured from the Bactrim group were 1 case of MRSA, and 1 case of combined Citrobacter freundii and methicillin sensitive Staphylococcus aureus (MSSA).

**Conclusion:**

There was no statistically significant difference in SSIs when Bactrim was given for two weeks after surgery. However, two subjects who developed infection from the Bactrim group were paradoxically affected by gram-negative and antibiotic resistant organisms. So, clinicians should be judicious in their use of oral antibiotics after spine surgery. Level of Evidence: III

## Introduction

Surgical site infections (SSI) are a potential complication after spinal surgery. The infection rates of spinal surgeries reported in the literature range from 0.7 to 11.9% depending on the diagnosis and the complexity of the procedure [1], [2], [3]. SSI account for enormous medical, social, and economic costs for patients as well as hospitals [4], [5]. Direct costs include a longer hospital stay, additional procedures to eradicate the infection, and antibiotics. A postoperative infection may also have an emotional impact on a patient's view of the overall outcome, despite a generally successful treatment of the infection.

The evidence suggests that systemic intravenous antibiotic prophylaxis reduces the risk of postoperative infections [6], [7], [8]. The current antibiotic prophylaxis regimen is to give a first generation Cephalosporin one hour prior to surgery and to continue it for 24 hours after surgery, which is supported by North American Spine Society's clinical guidelines [20]. Vancomycin is indicated in high-risk patients carrying methicillin-resistant *Staphylococcus aureus* (MRSA) [6]. Clindamycin is generally used when patients are unable to have a first generation Cephalosporin because of allergies. This regimen is very good for gram-positive organisms, but it does not provide adequate coverage for gram-negative organisms [9], [10], [11].

Bactrim (sulfamethoxazole/trimethoprim) is a very effective and popular treatment for gram-negative and MRSA infections. Therefore, Bactrim has been studied as a post-operative prophylactic oral antibiotic regime in multiple surgical specialties, such as vascular, colorectal, and neurosurgery [17], [18], [19]. The objective of this study is to look for the association of the post-operative prophylactic use of Bactrim and the gram-negative surgical site infection after lumbar spine surgery.

## Material and methods

The current study is an IRB approved retrospective evaluation of patients undergoing lumbar spinal surgery between August 2010 and December 2019 at the University of California Irvine.

A retrospective review of 595 patients presenting with spinal diseases requiring surgery was performed. This included patients with disc herniations, stenosis, spondylolisthesis, and spinal deformity. All consecutive electronic charts were independently reviewed, and patients who underwent lumbar microdiscectomy, laminectomy, decompression with posterior instrumented fusion, posterior lumbar interbody fusion, or transforaminal lumbar interbody fusion were included in the study. All patients had a standard chlorhexidine prep with 2 gm of IV cefazolin given within an hour before incision and continued for 24 hours after. Patients who were allergic to cefazolin were given vancomycin 1 gm IV one hour prior to the procedure and continued 24 hours after surgery. Primary data recorded include their initial diagnosis, procedure performed, whether or not patients were given Bactrim after surgery. Patients who were given Bactrim were given Bactrim DS one tablet PO BID for 14 days. Secondary data collected included age, gender, medical comorbidities, and social history, such as smoking status and alcohol use.

If a wound infection was suspected based on clinical examination and laboratory results (CBC with differential, ESR, CRP), then the wound was explored under general anesthesia. Aerobic, anaerobic, AFB, and fungal cultures were obtained. The wounds were classified and treated according to the depth of infection. Superficial infections involved the superficial skin or subcutaneous tissues and were treated with local wound care and 5 to 7 days of oral antibiotics. Deep wound infections were those involving the subfascial layers and the spinal instrumentation and treated with serial surgical debridement IV antibiotics, and consultation with Infectious Disease specialists.

### Statistical methods

A Chi-square test was used to determine if there was a significant difference in infection rates between the Bactrim versus non-Bactrim groups, respectively. A Chi-square test was also performed for each secondary measure to see if there was a difference in the alcohol use, smoking status, or medical comorbidities between two groups. The alcohol use group was defined as patients who answered “yes” for “do you drink?” question in the social history. Patients documented with an active smoking status at the time of surgery was grouped as smoking group. A condition was considered a significant medical comorbidity if hypertension, diabetes, renal disease, coronary artery disease, or cancer was listed in the patient's past medical history. A logistic regression analysis was also performed to see if these factors were independent risk factors for developing infection after surgery. All hypothesis testing was performed with a level of significance of 0.05.

## Results

There were 511 patients in the control group where patients were not given any oral antibiotics after they had completed their 24 hours of post-operative IV antibiotics. There were 84 patients who received Bactrim after they had completed their 24 hours of post-operative IV antibiotics. The average age of the patients in the control group was 60.8 years old and 62.0 years old in the Bactrim group. In the control group there were 56.6% females and 43.4% males. In the Bactrim group there were 55.5% females and 44.5% males. Patient characteristics of both study groups are summarized in Table 1.

**Table 1:** Demographics of patients.

Characteristic	Bactrim Group	Control Group	
Mean age (years)	62.0	60.8	P = 0.69
Sex (female %)	55.5	56.6	
Smoker (N)	16	68	P = 0.52
Significant medical comorbidities (N)	5	39	P = 0.32
Alcohol use (N)	14	56	P = 0.48

### Infection rate

There were 11 infections out of 511 cases where no oral antibiotics were given (2.2%). There were 2 infections out of 84 cases where Bactrim was given (2.4%). Statistical analysis using Chi-square test was not statistically significant (P=0.89, [-0.030, 0.039]). All of these patients were successfully treated with irrigation and debridement and antibiotic treatment. The instrumentation did not need to be removed in any of the patients to successfully treat the infections.

### Organisms cultured

The organisms cultured from the no oral antibiotic group were 8 cases of methicillin sensitive Staphylococcus aureus (MSSA), 1 case of E. coli, 1 case of Pseudomonas aeruginosa, 1 case of MRSA (Table 2).

**Table 2:** xxx.

Organism	N
No Bactrim Group	
MSSA	8
MRSA	1
E. coli	1
P. aeruginosa	1
Bactrim Group	
MRSA	1
C. freundii + MSSA	1

The organisms cultured from the Bactrim group were 1 case of MRSA, and 1 case of combined Citrobacter freundii and methicillin sensitive Staphylococcus aureus (MSSA) (Table 2).

### Smoking

In order to normalize the groups as best as we could, we performed a chi square analysis to see if there was a disproportionate number of smokers between the groups (Table 1). There were no significant differences between the groups in regards to smokers (P=0.52, [-3.76, 3.92]).

In this study, smoking was noted to be a risk factor for developing a post-operative infection. The difference in the infection rates between smokers and non-smokers as a risk factor for post-operative infections was statistically significant (P<0.00001).

### Medical Comorbidities

A chi square analysis was performed to see if there were a disproportionate number of patients with diabetes and medical comorbidities between the groups. There were no significant differences between the groups in regards to patients with medical comorbidities (P=0.32, [-0.538,0.572]).

In this study, the difference in the infection rates between patients with medical comorbidities and those without was not found to be statistically significant but it did approach significance (P=0.07)

### Age

A Student T-test analysis was performed to see if there was a difference in age between the groups. There were no significant differences between the groups in regards to age (P=0.69).

In this study, the difference in the infection rates between patients older than 65 and those younger than 65 was not found to be statistically significant but it did approach significance (P=0.07).

### Alcohol

We performed a chi square analysis to see if there was a disproportionate number of who drank alcohol between the groups. There were no significant differences between the groups in regards to drinkers (P=0.48, [-4.38, 4.49]).

In this study, alcohol use was not a separate risk factor of infection. The difference in the infection rates between people who drank alcohol versus those who did not drink alcohol as a risk factor for post-operative infections was not statistically significant but it did approach significance (P=0.05)

## Discussion

SSI pose significant problems to patients and surgeons. Patients must undergo additional procedures to eradicate the infection, and this often results in additional pain, a longer recover time, and more days missed from work. These problems are even greater in patients who have posterior instrumentation placed. In patients who develop SSIs that have posterior instrumentation there is the added risk of loss of correction if the instrumentation must be removed, decreased rate of fusion, and even the risk of osteomyelitis [1], [2], [3]. Hence, it is to the patients’ and surgeons’ best interest to do everything that is reasonable to decrease the incidence of SSIs.

In a study by Al Farii et al., the authors performed a retrospective review of 989 patients [9]. The authors noted an infection rate of 2.43% with 54% of the SSIs growing gram negative or a combination of gram-negative and gram-positive organsims. The authors noted a link between gram-negative SSI and spine surgeries involving more than three levels and surgeries involving the sacrum. Therefore, the authors proposed that there might be a potential benefit of gram-negative prophylactic antibiotic coverage in patients where more than three levels are operated and surgeries involving the sacrum. In another study by Abdul-Jabbar et al., the authors also noted a 30.5% rate of gram-negative organisms in their SSI [10]. These authors noted “ gram-negative organisms accounted for a sizeable portion of SSI, particularly among lower lumbar and sacral spine surgical procedures”. In a similar study by Long, et al, the authors performed a retrospective review on 6727 patients [11]. The authors noted that Cephalosporin-resistant gram-negative infection was common at lumbosacral levels. And the authors suggested “novel approaches to prophylaxis and prevention should be prioritized in this population”.

One potential source of gram-negative organisms is urinary tract infections. And some studies show that the SSI with gram-negative organisms may have spread via hematogenous spread from a urinary tract infection. In a study by Núñez-Pereira et al, the authors evaluated the SSI risk of patients who had urinary tract infections after having spine surgery [12]. The authors noted that the urinary tract was the probable source of SSI by Gram-negative bacteria in 38% (8/21) of cases. In another study by Núñez-Pereira et al., the authors evaluated the use of individualized intra-operative prophylactic antibiotics based on the pre-operative urinalysis [13]. The authors noted a decrease in SSIs from 17% down to 6.27% when targeted antibiotics were used.

Bactrim is a very effective and popular treatment for urinary tract infections [14]. In a paper by Jancel et al, the authors noted that Bactrim is considered a standard therapy for acute and recurrent urinary tract infections because of its activity against the most common uropathogens and its low cost and tolerability. And they also noted that Fluoroquinolones should not be used as a first-line drug therapy except in communities wherein resistance to trimethoprim is greater than 10-20% or in patients with risk factors for resistance. Other studies also recommend using Bactrim as a first line treatment for potential urinary tract infections [15], [16].

However, the results of this study show that the addition of Bactrim for two weeks after patients had completed their 24 hours of IV antibiotics was not associated with reduced post-operative SSI rates. Furthermore, there was a trend toward gram-negative and antibiotic resistance in the Bactrim group compared to non-Bactrim group.

This is the first study evaluating the association between the use of Bactrim and gram-negative SSIs after lumbar spine surgery. There is a need to develop an antibiotic prophylaxis regimen that will be effective against gram-negative organisms because the literature shows that there is a trend towards more gram-negative SSIs in spine surgery. But this study fails to support Bactrim as an effective prophylaxis medication. The results of this study are similar to the findings in the study by Núñez-Pereira et al. [12] In that study, the authors were looking for a potential relationship between postoperative UTI and SSI following posterior spinal instrumented fusion. Among patients who developed both postoperative UTI and SSI, patients who received ciprofloxacin for their UTI treatment had a higher rate of developing fluoroquinolone-resistant SSIs (46.13%) than those without ciprofloxacin (21.9%).

In conclusion, out study shows that the use of Bactrim as postoperative prophylactic antibiotic regime is not associated with a decreased rate of SSI after lumbar spine surgery. Clinicians should be judicious in their use of prophylactic oral antibiotics after spine surgery. There are many limitations to this study. First, the biggest confounder of this study is the criteria of using Bactrim. There was no guideline or consensus among surgeons when to give Bactrim to patients after surgery. Second, this is a one-center study including only five surgeons. Even though we analyzed nearly 600 patients, this study is underpowered given the incidence of infection of 2.2%. When we take into account the need of approximately 50 events in the control group to have an 80% power of detecting a 50% relative risk reduction, approximately 2700 patients would have been included in the control group [21,22]. Therefore, the results may be more variable with a larger number of hospitals and more surgeons. Third, this is a retrospective review. With a prospective, randomized study it may be possible to stratify patients better. However, we did a chi-square analysis and there was not a statistically significant difference between the groups in regards to infection risk factors such as smoking, medical co-morbidities, and alcohol use.

## Declaration of Competing Interest

The authors declare that they have no known competing financial interests or personal relationships that could have appeared to influence the work reported in this paper.
